# TGF-β: an active participant in the immune and metabolic microenvironment of multiple myeloma: TGF-β in the microenvironment of multiple myeloma

**DOI:** 10.1007/s00277-024-05843-4

**Published:** 2024-06-20

**Authors:** Han-Yue Xue, Fang Wei

**Affiliations:** 1 The First Clinical Medical College of Shanxi Medical University, 56 Xinjian South Road, Yingze District, Taiyuan, Shanxi People’s Republic of China; 2 Department of Hematology, The First Hospital of Shanxi Medical University, 85 Jiefang South Road, Yingze District, Taiyuan, Shanxi People’s Republic of China

**Keywords:** TGF-β, Multiple myeloma, Immunity, Metabolism, Tumor microenvironment

## Abstract

Although substantial quantities of potent therapies for multiple myeloma (MM) have been established, MM remains an incurable disease. In recent years, our understanding of the initiation, development, and metastasis of cancers has made a qualitative leap. Cancers attain the abilities to maintain proliferation signals, escape growth inhibitors, resist cell death, induce angiogenesis, and more importantly, escape anti-tumor immunity and reprogram metabolism, which are the hallmarks of cancers. Besides, different cancers have different tumor microenvironments (TME), thus, we pay more attention to the TME in the pathogenesis of MM. Many researchers have identified that myeloma cells interact with the components of TME, which is beneficial for their survival, ultimately causing the formation of immunosuppressive and high-metabolism TME. In the process, transforming growth factor-β (TGF-β), as a pivotal cytokine in the TME, controls various cells’ fates and influences numerous metabolic pathways, including inhibiting immune cells to infiltrate the tumors, suppressing the activation of anti-tumor immune cells, facilitating more immunosuppressive cells, enhancing glucose and glutamine metabolism, dysregulating bone metabolism and so on. Thus, we consider TGF-β as the tumor promoter. However, in healthy cells and the early stage of tumors, it functions as a tumor suppressor. Due to the effect of context dependence, TGF-β has dual roles in TME, which attracts us to further explore whether targeting it can overcome obstacles in the treatment of MM by regulating the progression of myeloma, molecular mechanisms of drug resistance, and various signaling pathways in the immune and metabolic microenvironment. In this review, we predominantly discuss that TGF-β promotes the development of MM by influencing immunity and metabolism.

## Introduction

Multiple myeloma (MM) is a malignant hematological disease with unlimited proliferation of plasma cells (PCs), accounting for 10% of hematological malignancies, and is the second most common hematological tumor after lymphoma [1]. Although considerable progress has been made in recognizing the accessibility of new treatment methods, most patients still display low or no response to drugs, making MM patients more likely to be converted to relapsed/refractory groups [2, 3]. Myeloma cells exist in the bone marrow microenvironment (BMME) and the interaction between them and components of the BMME is considered to be the key to the pathogenesis of MM [4]. TGF-β exists as a soluble element in the TME and is a multifunctional cytokine. Previous studies have demonstrated that TGF-β played an important role in the growth and metastasis of numerous tumor types, angiogenesis, drug resistance, anti-inflammation, immune regulation, lactate shuttle, and the balance of osteoclasts (OCs), and osteoblasts (OBs). Although it can be either a tumor suppressor or a tumor promoter, it’s believed to be involved in the pathophysiology and progression of MM [3]. Currently, there is expanding interest in using TGF-β as the therapeutic target in cancers involving MM. Hon Jo and Allison’s discovery of immune checkpoint inhibitors (ICIs) gained the Nobel Prize in 2018. TGF-β can also regulate immune checkpoint receptors on immune cells [5]. In the process of treating MM, ICIs are currently being developed, including anti-TGF-β antibodies and TGF-β inhibitors. At the same time, Chimeric Antigen Receptor (CAR)-T cell therapy has been applied in cancer immunotherapy and is attempting to be applied in combination with TGF-β inhibitors [6]. If the impact of TGF-β on the immune and metabolic microenvironment in MM can be more clearly defined, it’s expected to overcome the prevailing dilemma of treating MM, boost the remission rate, and decrease the recurrence rate of MM [3, 7].

## Multiple myeloma (MM)

MM is a cancer of PCs, marked by their uncontrolled growth in the BM and production of abnormal immunoglobulins [8]. MM mainly affects middle-aged and elderly individuals, with a higher prevalence in men than women. In China, the current prevalence rate is approximately 1/100,000, lower than in Western countries but increasing annually. Zhong, Hao, Zhang, et al. created a single-cell transcriptome atlas of the BM for MM patients staged by the Revised International Staging System (R-ISS), revealing new PC clusters with clinical importance. These findings aid in identifying potential immunotargets for MM, emphasizing the importance of considering MM’s various stages [9]. R-ISS, Durie Salmon (D-S), and ISS are MM’s clinical staging standards, all categorized into stages I, II, and III according to severity. D-S also differentiates A and B subtypes by renal function.

The etiology of MM remains unclear. Evidence suggests molecular cytogenetic abnormalities play a role in its development [10–12]. Factors such as radiation, viruses, and certain chemicals increase risk. Genetic correlation exists, with higher risk among immediate relatives [13, 14]. The symptoms of MM are diverse but typically include elevated blood calcium, renal insufficiency, anemia, and bone destruction (CRAB). Other symptoms include abnormal sensation, hepatosplenomegaly, swollen lymph nodes, fever, and so on. Recently, proteasome inhibitors (PIs), immune-modulatory drugs (IMIDs), and the application of autologous stem cell transplantation (ASCT) have enhanced the remission rate of patients with MM. However, some patients resist these drugs and experience relapse or progress [13, 15–17]. We need to explore novel therapeutic approaches to address these issues. Immunotherapy involves antibody-mediated immunotherapy, such as monoclonal antibodies (mAbs) targeting CD38 [18, 19], PD-1/PD-L1, CTLA-4 as well as TGF-β [19], and Bispecific antibodies (BiTEs) targeting CD3, CD138, and B-cell maturation antigen (BCMA) [19–21]. Cellular immunotherapy, primarily CAR-T, aims to modify T cells outside the body for targeted tumor control, with a promising CAR-T therapy for BCMA in MM [22]. Additionally, targeting metabolic pathways like lactate shuttle, glutamine synthesis, and remodeling bones in the TME can enhance progression-free survival (PFS) and overall survival (OS) rates of MM patients [19, 23, 24].

## The immune and metabolic microenvironment in MM

Myeloma cells thrive in the BM, highlighting its crucial role in supporting their survival. TME comprises hematopoietic and nonhematopoietic cells (bone structural cells, immune cells, and the vasculature), soluble factors (cytokines, growth factors, and adhesion molecules), and extracellular matrix (ECM), each contributing to multiple interconnected functions in the development of MM [8].

Bone marrow stromal cells (BMSCs) secrete TGF-β, promoting myeloma cell survival and osteolytic bone diseases by expressing Dkk1 [25]. This fosters an immunosuppressive microenvironment, shielding myeloma cells from T-cell-mediated cytotoxicity and perpetuating a harmful cycle [25, 26]. Andrea et al. observed a decline in potent effector KLRG1-NK cells in the BM during MM progression, linked to an altered chemokine microenvironment [27]. Notably, MM-BM mesenchymal stromal cells (BMMSCs) interactions negatively impact immunotherapeutic approaches, such as T cells, CAR-T cells, and Daratumumab-redirected NK cells, by increasing antiapoptotic proteins in MM [28]. Yu Huan Zheng et al. found that CD68 + macrophages in the BM shield myeloma cells from apoptosis induced by drugs and spontaneous processes by inhibiting caspase-dependent apoptotic signaling [29]. Gregory Whitehill et al. reported that elevated levels of CD11b + CD14- HLA-DR-/lowCD33 + CD15 + myeloid-derived suppressor cells (MDSCs) were observed in both peripheral blood and BM compared to healthy controls. MDSCs promote MM growth while inhibiting T-cell immune responses. Conversely, MM cells stimulate MDSC growth from healthy donor cells, revealing a reciprocal interaction [30]. Kim De et al. identified that Cancer-Associated Fibroblasts (CAFs) produce cytokines, chemokines, and pro-inflammatory factors to foster MM growth and metastasis [31].

Cytokines, particularly interleukin, play a crucial role in the soluble components of the TME, secreted by various cells of the TME. Interleukin, in conjunction with TGF-β, fosters a supportive TME for myeloma cell survival. BMSCs produce both interleukin-6 (IL-6) and TGF-β, with the latter influencing the former’s secretion [32]. IL-6 overexpression is associated with bone diseases like metastatic cancers, contributing to bone lesions and drug resistance [33]. Teramachi et al. discovered that co-culturing myeloma cells with BMSCs promotes TGF-β-activated kinase-1 (TAK-1) and elevates vascular cell adhesion molecule-1 (VCAM-1) levels in BMSCs. This interaction strengthens MM and BMSC adhesion, triggers IL-6 secretion, and increases nuclear factor κ-B ligand (RANKL) expression in BMSCs. Consequently, inhibiting TAK1 disrupts MM-BMSC adhesion, inhibits osteoclast (OC) genesis, and may revive osteoblastic activity [4]. Melanie Werner-Klein et al. determined that the IL-6/PI3K pathway plays a significant role in activating disseminated cancer cells (DCCs) despite their lack of membrane-bound IL-6 receptors, with IL-6 trans-signaling prompting a stem-like state in mammary epithelial cells through gp130 [34]. Further research by Matthew Ho and his team demonstrated that disabling Histone deacetylase 3 (HDAC3) via knock-out (KO), knock-down (KD), or pharmacological methods in BMSCs reduces myeloma cell proliferation, effectiveness confirmed in patient-derived myeloma cell cultures with BMSCs [35]. Furthermore, Alissa Visram et al. reported that marked downregulation of interferon, TGF-β, IL-6, and TNF-α signaling pathways in abnormal plasma cells from patients with triple-refractory multiple myeloma (TRMM) compared to newly diagnosed or relapsed cases, indicating lower responsiveness of TRMM to new immunotherapies [36, 37]. Ming Chen et al. reported that the frequency of CD4 + PD-1+, CD8 + PD-1+/LAG-3+, as well as IL-6, IL-17, and TNF-α were found as risk factors for the incidence of relapsed/refractory MM (RRMM) in all MM [38]. Particularly, IL-6 and TGF-β not only influence the treatment outcomes but also the prognosis of MM. Further research has linked IL-10 and IL-18 with adverse prognosis in MM [39, 40]. Moreover, interleukins like IL-2 interact with TGF-β in immune signaling; IL-2, along with TGF-β produced by tumor cells, promotes the generation of regulatory T cells (Treg), which generally counteract anti-tumor immunity. However, the presence of IL-21, when combined with IL-2, encourages expansion of T cells, especially naïve CD4 + cells, and can specifically inhibit Treg proliferation by promoting non-Treg cell growth during early cell activation phases [41].

Cytokines, growth factors (GFs), and adhesion molecules are key players in the TME, contributing to interactions between tumor cells and their surrounding milieu. This dynamic isn’t unique to solid tumors but is also observed in various hematologic malignancies, such as MM [42]. Vascular endothelial growth factor (VEGF) not only stimulates angiogenesis, aiding myeloma cell nourishment and subsequent proliferation and spread but also contributes to bone degradation [42]. Exosomes, produced by stem cells and acting as GFs, are polypeptides that govern cell proliferation and other functions by attaching to specific cell membrane receptors. Jinheng Wang et al. discovered that exosomes from murine myeloma carry angiogenesis-promoting proteins that boost angiogenesis and endothelial cell proliferation [43]. Addressing the harm of excess vascular growth in myeloma, Boris Lin et al. identified PTK787/ZK 222,584 (PTK787), a molecule that targets the VEGF receptor’s tyrosine kinase to thwart angiogenesis. PTK787 not only augments dexamethasone’s myeloma cell growth inhibition but also circumvents IL-6’s protective effect against dexamethasone-induced cell death. Additionally, PTK787 impedes VEGF-stimulated myeloma cell migration and reduces MM cell proliferation and IL-6 and VEGF release when these cells are attached to BMSCs [44]. Interaction between myeloma cells and BMSCs is central to MM progression and organ damage. Yu-Tzu Tai et al. demonstrated that the B-cell activating factor (BAFF) (0-100 ng/mL) enhances MM cell adhesion to BMSCs in a concentration-dependent manner, mediated by AKT and NF-κB signalings [45]. Similarly, Sonia D’Souza’s research revealed that stromal and osteoclast-derived annexin II (AXII) increases MM cell attachment and growth through its receptor on MM cells, activating ERK1/2 and AKT pathways [46]. Klaus Podar’s work with the selective-adhesion molecule (SAM) inhibitor Natalizumab, an anti-integrin-α4 monoclonal antibody, showed it prevents MM cell adhesion to the TME, disrupts pre-bound MM cells, inhibits VEGF-induced angiogenesis, impedes MM cell migration, and resensitizes MM cells to bortezomib [47].

ECM is recognized as advantageous for myeloma cell proliferation and persistence. Proteomics reveals that both MM patients and healthy individuals exhibit a diverse range of ECM protein outputs from the BM fibroblasts, potentially nurturing myeloma cell growth and contributing to drug resistance. Furthermore, increased soluble syndecan-1 (CD138) levels, which are associated with myeloma cells, have been linked to poor prognoses [48].

## Transforming growth factor-β (TGF-β)

The human TGF-β superfamily comprises 33 structurally similar members, including TGF-β isoforms (TGF-β 1/2/3), Bone Morphogenic proteins (BMPs), Growth and Differentiation Factors (GDFs), activins, nodal, inhibin, and AMH/MIS, all dimeric secreted peptides [49]. This family is bifurcated into two categories: the TGF-β/activin/nodal and BMP/GDF/AMH subfamilies, differentiated by sequence, structural traits, and signaling pathways. TGF-β is produced by multiple cells within the TME, with reports of its secretion in various cancers such as colon [50], breast [31], and MM [51, 52]. Immune cells, notably Foxp3 + Tregs and IL-10 + regulatory B cells (Bregs), also serve as sources, with the former inhibiting anti-tumor immunity in MM and the latter reducing T cell proliferation [14, 51, 53, 54]. Additionally, M2-like tumor-associated macrophages (TAMs) are identified as significant contributors to TGF-β production [31, 49, 51, 55–57]. Osteocytes, containing osteogenic cells, osteoblasts (OBs), and OCs, contribute to bone health. Under normal conditions, osteogenic cells transform into OBs, which generate bone tissues through synthesis, secretion, and mineralization. OCs are involved in bone resorption, breaking down bone matrix and inactive tissue. In MM patients, however, there is significant bone destruction due to an imbalance in bone formation and resorption, largely stemming from altered RANKL/osteoprotegerin (RANKL/OPG) ratios. The interaction between OBs and MM cells inhibits OB formation, influenced by soluble factors like osteocalcin, osteopontin, and TGF-β [53]. TGF-β, released during bone resorption, further impedes OB differentiation [58]. MM also promotes neovascularization to support myeloma cell metastasis, driven by VEGF and TGF-β from BMSCs and PCs [53]. Additionally, TGF-β1 from platelet α-granules might play a significant role in MM, with lower serum levels observed in thrombocytopenic patients, indicating platelets as its primary source [59, 60].

Derived from diverse sources, TGF-β is a multifunctional cytokine involved in various physiological and pathological processes, which requires release from the ECM to become active [56]. Normally stored as a potential complex, TGF-βs are synthesized as prohormones, consisting of a signal sequence, a large N-terminal latency-associated peptide (LAP) that prevents interaction with receptors, and a short C-terminal segment representing the mature, bioactive cytokine. In the Golgi apparatus, TGF-β dimerizes and is cleaved by furin. Post-cleavage, the bioactive cytokine, and LAP remain non-covalently bonded forming latent TGF-β (L-TGF-β), which inhibits signaling due to the LAP function. Frequently, LAP links with L-TGF-β-binding proteins (LTBPs), forming the large L-TGF-β complex (LLC) retained in the ECM by interaction with glycoproteins like fibrillin. Alternatively, in specific cells, L-TGF-β binds to either GARP (LRRC32) or LRRC33 post-furin cleavage, facilitating membrane loading and TGF-β release and signaling regulation. GARP is expressed in tumor cells, Tregs, endothelial cells, and platelets whereas LRRC33 is found in macrophages and microglia [49, 57, 60]. Similar to TGF-β storage mechanisms, its release from the ECM is meticulously regulated by enzymatic and non-enzymatic activities. Initially, the ECM protein thrombospondin 1 binds to a specific sequence in LAP, preventing its association with active TGF-β and thus promoting the release of the latter. Additionally, various proteases, including plasmin, cathepsin D, and matrix metalloproteases (MMP), can cleave L-TGF-β and release active TGF-β. Beyond enzymatic actions, there’s strong evidence that integrin activities, particularly αVβ6 and αVβ8 integrins, are crucial, as they connect to the Arg-Gly-Asp (RGD) motif in the LAP domain of TGF-β1/3 with high affinity. Specifically, αVβ6 translates cytoskeletal tension into physical forces, pulling on the LLC, unfolding LAP, and discharging active TGF-β upon cellular contraction, relying on the attachment of the small latent complex to the ECM via LTBP1. Unlike αVβ6, αVβ8 targets L-TGF-β bound to GARP, with GARP serving as a chaperone orienting L-TGF-β for αVβ8 binding. This triggers a conformational change in L-TGF-β which activates TGF-β receptors while the ligand remains bound to GARP, without transmitting cellular contraction forces to the L-TGF-β molecule [49, 56, 57, 60].

Once liberated from the ECM, TGF-β ligands engage with two ubiquitously expressed, high-affinity transmembrane serine/threonine kinase receptors: type I (TGF-β R1) and type II (TGF-β R2) [54, 60]. TGF-β can also interact with these receptors through the co-receptor β glycan (βG, also known as TGF-β R3), which is critical for TGF-β2 association [57, 60]. Specifically, TGF-β1/2/3 exclusively binds to the TGF-β R1/R2 pairing [57]. The canonical TGF-β/SMAD signaling pathway encompasses a membrane-to-nucleus communication process activating SMAD transcription factors directly through receptors. SMAD proteins are categorized into three types: receptor-activated SMADs (R-SMADs: SMAD1/2/3/5/8), common-pathway SMADs (Co-SMADs: SMAD4/10), and inhibitory SMADs (I-SMADs: SMAD6/7), with SMAD2/3 activated by TGF-β receptors and SMAD1/5/8 by BMP receptors [57]. SMAD6/7 regulates TGF-β receptor degradation via recruiting SMURF1/2 ubiquitin ligases and counteracting ubiquitin-specific peptidases (USP11, USP15) and compete with the R-SMADs for receptor interaction, which creates feedback loops [57, 61]. SMAD proteins have MH1 and MH2 domains [57]. MH1 connects to DNA in R-SMADs and Co-SMAD, while MH2 associates with SMADs, transcription factors, and chromatin modulators [57]. TGF-β signaling initiates when TGF-β binds TGF-β R2, which then pairs with TGF-β R1, forming a tetrameric receptor complex that phosphorylates R-SMAD (e.g., SMAD2/3) at the C-terminal serine residues [54, 60]. The phosphorylated R-SMADs form complexes with SMAD4 or transcription intermediary factor 1 gamma (TIF1γ), migrate to the nucleus and influence transcription alongside factors such as Runx1, E2F, coactivators, and corepressors [54, 57]. Within the nucleus, CDK 8/9 phosphorylates R-SMADs, allowing more co-factor binding and setting the stage for further phosphorylation by glycogen synthase kinase 3 β (GSK3 β), signaling SMAD ubiquitination and degradation by ligases like NEDD4L and SMURF1. Concurrently, R-SMAD dephosphorylation and DNA detachment facilitate signal reinitiation, underscoring the pathway’s effectiveness [57]. What’s more, activated TGF-β receptor complexes can trigger lesser-known, SMAD-independent pathways, such as mitogen-activating protein kinases (MAPKs), Rho-like GTPase, phosphatidylinositol-3-kinase/AKT, TNF receptor-associated factor 4/6 (TRAF4/6), and NF-κB pathways, etc. These pathways modulate a wide range of functions across varied cellular and tissue contexts [31, 54, 57, 61, 62].

## TGF-β in the immune microenvironment in MM

The interaction between myeloma cells and various components in the TME often leads to the proliferation, survival, and metastasis of myeloma cells, excessive angiogenesis, bone destruction, drug resistance, and most importantly low anti-tumor immunity, including innate immunity and acquired immunity, in which the immune cells play a major role [49]. However, TGF-β changes the effect that immune cells should play under normal conditions through various signaling pathways, mainly resulting in the decreased function of effector immune cells and ultimately forming an immunosuppressive microenvironment [49, 51, 54, 56, 57]. Acquired immune cells involve T cells, including naive, effector, and memory T cells, and subsets like helper, regulatory, and cytotoxic T cells [51], as well as antigen-presenting cells (APCs), such as dendritic cells (DCs) and B cells. APCs are divided into dedicated APCs and non-dedicated APCs. The former contains MHCII, such as dendritic cells (DCs) and B2 cells and the latter includes vascular endothelial cells, epithelial cells, stromal cells, skin fibroblasts, activated T cells, etc. Innate immune cells include phagocytes, NK cells, and B1 cells [51, 57]. Phagocytes consist of monocytes that are precursors of macrophages and DCs and macrophages stimulated by special inducible factors. NK cells can directly kill cells without relying on antibodies and complement via the production of IFN-γ [56].

### TGF-β and acquired immune cells in MM

Naïve T cells: They originate and mature in the thymus before migrating to peripheral lymphoid organs, and are unresponsive until activated by antigens to differentiate into various subtypes such as Th cells, Tregs, and CTLs [51]. TGF-β, however, inhibits their development into effector cells [60], with research by Rana et al. indicating that naïve T cells downregulate TGF-β receptors to diminish its signaling [51]. This cytokine skews differentiation, decreasing functional Th1 and Th2 cells while increasing Th17 cells, which secrete tumor-promoting cytokines IL-17 and IL-22 [49]. Moreover, TGF-β from DCs prompts the development of Foxp3 + Tregs that can suppress antigen-specific T cell expansion and foster the generation of less responsive memory T cells by inhibiting crucial TCR-CD28 signaling required for effector T cell formation [51, 54, 56].

Effector T cells: They derive from T cells after antigenic stimulation, encompass various types including Th cells, Tregs, and CTLs, and function by secreting lymphokines. During this process, a subset transitions into memory T cells—this is known as the induction stage. The rest then engage with target cells to induce apoptosis through granule exocytosis, with perforin creating pores in the target cell membranes to execute cytotoxicity [54]. Effector T cells also emit immunomodulatory molecules like interleukins and interferons. Nonetheless, in MM, TGF-β hinders the growth, differentiation, and functional activity of CD4 + and CD8 + effector T cells [51, 54, 56]. Daniele V. F. Tauriello et al. reported that increased TGF-β in the TME represents a primary mechanism of immune evasion that promotes T-cell exclusion and blocks acquisition of the Th1-effector phenotype [50]. TGF-β blocks IL-2 production, thereby inhibiting IL-2-dependent effector T cell proliferation and maturation [60]. It also suppresses Ca2 + influx-triggered TCR signaling, Tec kinase activation, NFAT translocation, and MAPK/ERK pathway activation, impeding effector T cell differentiation [54, 56, 57, 63]. TGF-β impacts CD4 + effector T cells by inhibiting nuclear κ B family protein, which dampens IFN-γ expression [51] and reduces key transcription factors like T-bet, GATA-3, and STAT-4 crucial for Th1 and Th2 cells [56, 57]. For CD8 + effector T cells, TGF-β boosts CD39 and CD73 [56], while IL-17 receptor signaling monitors TCR signaling duration, affecting CD8 + T cell differentiation [51, 54]. CD8 + effector T cells employ cytotoxicity via granulocyte exocytosis (perforin, Granzyme A/B) and Fas-Fas ligand pathways, along with TNF-α and IFN-γ secretion for target cell elimination [64]. However, TGF-β-activated SMADs and ATF1 block various genes crucial for CD8 + T cell cytotoxicity, including perforin, Granzyme A/B, FasL, and IFN-γ [51, 54, 56, 57]. TGF-β also targets key regulators like T-bet, EOMES, and BLIMP-1, which promote cytotoxic molecule expression. However, TGF-β suppresses T-bet, EOMES, and BLIMP-1, hindering effector T cell functions. Tumor-derived TGF-β induces miR-23α to inhibit BLIMP-1 [54]. Furthermore, TGF-β boosts Zeb1, supporting memory T cell survival, while suppressing Zeb2, favoring effector T cell differentiation. Low Zeb2 levels result in decreased Bcl-2 expression, an anti-apoptotic molecule in effector T cells [54].

Memory T cells: In normal conditions, the body uses memory methods to combat antigens upon subsequent invasions, effectively destroying target cells. However, in the presence of tumors like MM, TGF-β not only hampers Th1 cell cytotoxic activity and biases T cell differentiation towards Th2 phenotype but also fosters hypo-responsive memory T cells by inhibiting TCR-CD28 signaling [56]. Furthermore, TGF-β decreases levels of T-bet, EOMES, and BLIMP-1 [54], and can convert effector T cells into tissue-resident memory T cells. TGF-β signaling promotes T cell memory retention in epithelial tissues by increasing αE (CD103) and β7 integrin subunits levels, independently of the TGF-β/SMAD pathway. Studies show TGF-β increases CD8 + CD103 + T cells, expressing immunosuppressive molecules (CTLA-4 and IL-10), aiding tumors in immune evasion [49, 54]. It also prevents memory T cells from leaving secondary lymphoid organs by downregulating KLF2, causing the decrease of sphingosine-1-phosphate receptor 1 (S1P1) expression and ultimately establishing T-cell retention in tissues [54].

Th1 cells: They combat intracellular bacteria and protozoa, primarily stimulated by IL-12, and include killer CD8 + T cells, IFN-γ-secreting CD4 + T cells, IgG-producing B cells, and macrophages. However, in the TME, there’s elevated TGF-β, reduced Th1 activity, and diminished immune cytotoxicity [50]. Studies have confirmed that naïve T cells cultured with TGF-β couldn’t differentiate into the Th1 phenotype [57]. TGF-β inhibits CD4 + Th1 cell proliferation by suppressing IL-2 expression via the TGF-β/SMAD signaling pathway, mediated by TOB1 [49, 51, 57]. It also hampers Th1 differentiation by downregulating STAT4 and T-bet, crucial transcription factors, thus hindering IFN-γ production [49, 57]. Moreover, in MM, TME is immunosuppressive, marked by fewer effective anti-tumor immune cells and more apoptotic cells due to TGF-β. Furthermore, TGF-β upregulates CDNK1A (p21Cip1) and CDNK1B (p27Kip1) but downregulates MYC, which are all downstream cell-cycle regulators, identifying the effect of TGF-β [49, 57, 61].

Th2 cells: They are a subset of CD4 + T cells, and produce cytokines like IL-4/5/10/13, stimulating Th2 cell growth and suppressing Th1 cell development. Their primary impact lies in bolstering B cell proliferation for humoral immunity [49]. Th1/Th2 cells maintain equilibrium but may shift, termed “Th1/Th2 drift”, potentially leading to diseases, including cancers [65]. TGF-β influences Th2 cells by impeding activation through TCR signaling inhibition and downregulating crucial transcription factors like T-bet and GATA3, pivotal for Th2 cell differentiation [49, 54, 56, 63]. Tauriello et al. found that SOX4, a TGF-β target, blocks Th2 cell transcription factors [49]. TGF-β also hampers Th2 cell differentiation by inhibiting Tec kinase Itk, reducing Ca2 + influx [63]. Chen et al. suggest TGF-β inhibits Th2 cell development but not activation [63], and some tumors show heightened Th2 cell gene activity [49]. Overall, TGF-β still weakens the role of Th2 cells in anti-tumor immunity [49, 63].

Th9 cells: Th9 cells, a recently identified group of CD4 + Th cells marked by PU.1 [54], contribute to various health conditions through IL-9 cytokine expression. The interaction of TGF-β and IL-4 signaling pathways promotes the expression of PU.1 and IL-9 production, resulting in both inflammatory and anti-tumor effects. Moreover, IL-4 suppresses TGF-β-Id3 expression through TAK1 activation, which is crucial for Th9 cell differentiation [51]. The pre-clinical model found that activating glucocorticoid-induced TNFR-related protein (GITR) on T cells triggers anti-tumor effects via Th9 response. Besides MM resistance, Th9 cells and IL-9 induce myeloma cell apoptosis, support T cell survival, activate mast cells, and boost IFN-γ production by T and NK cells [54]. Elevated Th9 levels correlate with better prognosis, suggesting potential therapeutic targeting of Th9 and IL-9.

Th17 cells: They are a recent CD4 + T cells subset, that produce IL-17 A and IL-22, crucial in autoimmune diseases and body defense [49]. TGF-β, IL-6, IL-21, and IL-23 aid Th17 cell formation, while IFN-γ, IL-2, IL-4, and Socs3 inhibit it. TGF-β boosts Th17 differentiation [51] and steers naïve CD4 + T and Th1 cells towards Th17 [49]. Non-canonical TGF-β signaling, including AKT, MAPK, and NF-κB pathways, drive Th17 differentiation [54]. TGF-β, with IL-6 or IL-21, heightens RORγt levels, governed by RORC, crucial for Th17 differentiation [54]. TGF-β and IL-6-induced Th17 cells, with high aryl hydrocarbon receptor (AhR) levels, secrete IL-10, exhibiting immunoregulation [54]. TGF-β can regulate Th17 cells by inducing adenosine production, suppressing immune responses, and modulating T-bet and CD39/CD73 expression [54]. Inhibition of TGF-β reduces Th17 cell formation and promotes IFN-γ production by CD4 + T cells. Additionally, high TGF-β concentrations inhibit IL-23R expression, leading to Foxp3-mediated suppression of RORγc expression, favoring Tregs over Th17 cells [54].

Regulatory T cells (Tregs): They are a subset of T cells, that regulate the body’s autoimmune response, categorized as natural (n-Tregs) and induced adaptative (i-Tregs) [51, 54]. In MM’s TME, elevated TGF-β and IL-6 levels prompt CD4 + Tregs to inhibit immune surveillance and reduce effector T cells [8, 65]. Tregs secrete IL-10, fostering MM development [53], while IL-10 activates TGF-β [19]. Higher IL-10 levels and increased Tregs correlate with shorter patient survival [8, 53]. TGF-β and IL-2 induce Foxp3 expression in CD4 + T cells, suppressing effector T cell expansion [51, 54, 56, 57, 60, 66], contingent upon stable Foxp3 expression for Tregs’ immunosuppressive function. In the thymic, non-coding sequences-2 (CNS2) region near Foxp3’s transcriptional starting site, significant demethylation ensures stable Foxp3 expression. However, induced Tregs in the periphery exhibit less demethylation and often lose Foxp3 expression. TGF-β-induced Tregs also lose Foxp3 stability, but certain factors like retinoic acid and CDK8/19 inhibitors can promote demethylation, enhancing Foxp3 expression [51]. Foxp3 enhancer enriches SMAD3, which interacts with NFAT [51, 57], and P300 acetyltransferases, linked to NF-κB and AP-1, positively regulate Treg generation [51]. L-TGF-β, associated with GARP on the cell membrane, induces Th17-to-Treg trans-differentiation [49, 57]. Additionally, TGF-β and prostaglandin E2 trigger this process [57]. TGF-β protects Tregs and their precursors from apoptosis in the thymic environment, generating more immunosuppressive Tregs in the TME of MM [51].

Cytotoxic T lymphocytes (CTLs): CTLs are specific T cells that secrete cytokines and directly attack tumor and virus-infected cells. They play a crucial role in antiviral and antitumor immunity alongside NK cells. Upon encountering target cells, CTLs release perforin, forming pores in the target cell membrane, and GzmB, which induces apoptosis [64]. However, immunosuppressive elements like MDSCs and M2-TAMs in the TME of MM often hinder CTLs and NK cells. TGF-β is known to inhibit CTLs [54, 65]. TGF-β inhibits TCR signaling and suppresses Myc and Jun genes, halting CTL proliferation [49]. It also hampers CTL differentiation and activation by targeting master regulators like T-bet, EOMES, and BLIMP-1 [49, 54]. Moreover, TGF-β blocks CTL migration into MM by silencing C-X-C chemokine receptor 3 (CXCR3) genes [49]. It mainly promotes tumor progression by dampening CTL effector function and downregulating genes encoding effector molecules [54, 57]. Furthermore, TGF-β suppresses EOMES, which is required to establish the gene program of effector CTLs, causing immune evasion [57]. The canonical TGF-β/SMAD pathway, along with ATF1, suppresses genes crucial for CTL lytic functions, impairing their ability to kill myeloma cells [49, 54, 57, 64].

Dendritic cells (DCs): DCs arise from the BM stem cells, differentiating into myeloid DCs (MDCs or DC1) and lymphoid DCs (LDCs or DC2). They serve as potent APCs, pivotal in immune responses. Immature DCs migrate efficiently, while mature DCs activate T cells, orchestrating immune reactions. However, MDSCs and certain cytokines from myeloma cells like IL-6, IL-10, and TGF-β [19, 65]. TGF-β in the TME induces an immune-suppressive DC phenotype by upregulating ID1 via the TGF-β/SMAD pathway [49, 57]. Tumor-associated DCs also express αVβ8 integrin, promoting CD4 + T cell transformation into immunosuppressive Treg cells [49]. TGF-β affects chemokine receptor expression, hindering DCs’ entry into lymph nodes [49] and suppressing MHCII gene expression, reducing DCs’ antigen-presenting ability and T cell activation [49, 56, 57]. In MM models, TGF-β upregulates IDO in PC-like DCs (PDCs) and CCL22 chemokine in MDCs, leading to immune evasion [57].

Cancer-associated fibroblasts (CAFs): CAFs are integral components of the TME in MM, with their abundance increasing in more advanced tumors [16, 31, 57]. CAFs undergo morphological and functional changes, promoting MM growth and resistance to drug-induced apoptosis [16, 31, 49, 67]. They contribute to MM development by remodeling the ECM, regulating metabolism and angiogenesis, and interacting with myeloma and immune cells through the secretion of various factors, such as GFs, cytokines, and chemokines [67]. TGF-β is a key driver of the conversion of resident fibroblasts and BMMSCs in MM to CAFs through processes like mesenchymal transition (MT) [16]. Notably, endothelial cells, epithelial cells, as well as certain stem and progenitor cells can also transform into CAFs via signaling pathways like endothelial MT (EndMT) or epithelial MT (EMT), mediated by TGF-β [31]. CAF activity is characterized by heightened cytokine and GF production, notably VEGF, TGF-β, and HGF [31, 57, 67], in conjunction with αsma, which expedites CAF differentiation [31]. Interaction of TGF-β signaling with SNAIL and Zeb1/2 regulates EndMT [31, 67]. Conversely, SMAD7 binding to SMAD2/3 obstructs the EMT pathway, countered by TGF-β inducing miR21 maturation to decrease SMAD7 expression [31]. CAF-derived TGF-β impedes immune cell migration to tumor centers, fostering immune evasion, tumor progression, and metastasis [57, 67]. It has been reported that tumors rich in TGF-β-activated CAFs often had a low response to checkpoint immunotherapies along with poor prognosis [49, 57].

### TGF-β and innate immune cells in MM

Macrophages: Macrophages are highly adaptable cells that change phenotype based on environmental cues. They exist primarily as M1 (classical activated or inflammatory) and M2 (alternating or anti-inflammatory) subtypes [49]. In MM, the TME tends to steer macrophages towards the M2 subtype, known for its immunosuppressive role [57]. There are various connections between TGF-β and macrophages in the TME in MM. M2-like macrophages not only secrete TGF-β through integrin αVβ8 and MMP14 [56, 57] but also release TGF-β, inducing fibroblast activation indirectly [31, 55]. It has been reported that IL-10 produced by MDSCs could activate M2-like macrophages [19]. Besides, TGF-β can recruit M2-like macrophages, which can secrete immunosuppressive factors, such as IL-10 and TGF-β via the TGF-β/SMAD signaling pathway together with SNAIL [49, 51, 57]. What’s more, TGF-β/SMAD6/7 in macrophages suppress anti-inflammatory reactions alongside NF-κB. SMAD6, acting as an inhibitory factor, recruits E3-ubiquitin ligases, causing MYD88 polyubiquitination and sequestering adapter protein spelling-1, thus fostering inflammatory responses. This inhibition is crucial for immune evasion, MM growth, and drug resistance [19, 57]. Moreover, TGF-β boosts PD-L1 expression on M2-like macrophages, aiding immune evasion [49]. It also upregulates CXCR4, facilitating monocyte migration to tumor sites by inducing blood vessel permeability [57].

Natural killer cells (NK cells): NK cells play vital roles in immune responses, including combating tumors and viruses. However, in MM, the TME often impairs NK cell function, like NK cell exhaustion, desensitization, and exclusion through interactions with myeloma cells and molecules like TGF-β [19, 49, 65, 68]. NK cells mainly rely on their receptors, such as NKG2D, NKp30, and DNAM-1 to recognize tumors, but TGF-β downregulates or even impairs expression of these receptors, decreasing MM surface ligands recognition, cytotoxicity, and immunosurveillance [51, 56, 57, 69, 70]. Additionally, TGF-β can also decrease the expression of an adaptor of NKG2D, such as DAP12 by upregulating miR-183 [57]. Expect for the effect on receptors, the TGF-β /SMAD signaling pathway controls the production of IFN-γ and GzmB from NK cells in response to antibody-dependent cell-mediated cytotoxicity(ADCC) together with T-bet, impeding Th1 response, and which could be counteracted by inflammatory signals [49, 51, 56, 57, 69]. The TGF-β pathway impacts murine NK cell function and metabolism by inhibiting mTOR, independent of the canonical pathway [57]. However, Vanessa Zaiatz-Bittencourt et al. reported that in human NK cells, the canonical TGF-β pathway prevails in NK cells [71]. Finally, TGF-β can transform NK cells into type I innate lymphoid cells (ILCs), reducing immunosurveillance and upregulating CTLA-4 but downregulating IFN-γ [51]. It can also convert type II ILCs to type III, which produces IL-17, an immunosuppressive cytokine [49].

Neutrophils and myeloid-derived suppressor cells (MDSCs): Neutrophils, known as the natural candidates to perform in vivo medical tasks, are the most abundant and studied type of granulocyte [49]. As the first line of defense for the host to resist invading pathogens, it not only has the inherent phagocytic ability, which can absorb nanoparticles and phagocytic dead red blood cells, and after activation, clear foreign pathogens and load targets, but also can migrate across blood vessels to adjacent tissues. However, various tumors including MM exhibit increased numbers of neutrophils expressing metalloproteinases associated with poor outcomes [49, 57]. Also, Jackstadt, R. et al. reported that TGF-β2 enabled to recruit the of neutrophils. Similar to TAMs, neutrophils can also be divided into two phenotypes called N1 and N2. TGF-β often transforms neutrophils into the pro-tumorigenic N2 phenotype with decreased cytolytic activity and expression of pro-inflammatory cytokines, suggesting the failure of immune surveillance [49]. Except for neutrophils derived from the myeloid system, MDSCs also play an important regulatory role in tumor immunity. They are pathologically activated neutrophils and monocytes with strong immunosuppressive activity and adverse clinical outcomes. In the TME of MM, there are high levels of MDSCs [65]. They have significant effects on cancer immune invasion through increasing angiogenesis and formation of OCs, impairing DCs maturation, and suppressing the proliferation of CD4 + T cells [19, 53, 65]. TGF-β controls MDSCs differentiation and immunoregulatory activity, promoting MM progression and metastasis [49, 56]. The latest research describes some new genomic and metabolic features of MDSCs, which shape the specific functions of MDSCs and contribute to targeted therapies based on these cells, especially in cancers and autoimmune diseases.

## TGF-β in the metabolic microenvironment in MM

The unique TME in MM is a necessary condition for the occurrence, development, and metastasis of abnormal PCs, which is characterized by interdependence, mutual resistance, mutual struggle, and mutual promotion. Therefore, many researchers have taken steps to treat diseases by targeting the metabolic barrier [24, 72]. In addition to the immunosuppression described above, the TME also contains overall hypoxia, acidification, vascular high permeability, interstitial hypertension, and inflammatory reactivity. Firstly, MM has been reported to exist in hypoxic environments because myeloma tissues have a high demand for oxygen and other energy substances such as glucose and glutamine [65, 73]. As the blood supply to the tissue is insufficient, hypoxia occurs. In this case, TGF-β can activate more CAFs to promote the development of MM [31]. Secondly, myeloma tissues begin to breathe without oxygen due to hypoxia, causing lactic acid (LA) accumulation and acidification of the TME as a whole. Shogo Kumagai et al. reported that under low-glucose environments, Treg cells actively absorbed LA through monocarboxylate transporter 1 (MCT1) and promoted NFAT1 involved in TGF-β signaling pathway to translocate into the nucleus, which enhanced the expression of PD-1, whereas PD-1 expression by effector T cells was dampened [74]. Then, there is also high permeability of blood vessels, and MM blood vessels generally exhibit an irregular spiral shape with a certain increase in interstitial fluid and vascular viscosity. As mentioned above, the TGF-β signaling pathway can increase the extravasation and metastasis of myeloma cells through the high permeability of blood vessels. Additionally, interstitial hypertension is caused by the inability of MM to regulate the dynamic balance of tissue fluids and the high permeability of blood vessels. Finally, inflammatory reactivity and various chronic inflammations may occur in the later stages of MM. Jiadong Xia et al. performed a pan-cancer analysis comparing the activation of the TGF-β pathway among different TMEs based on multi-omics data and found that compared with non-inflamed tumors, increased TGF-β signaling activity appeared in four non-cancer cell types within TME in inflamed tumors [75].

In addition, an acid environment and increased glutamine are associated with bone destruction, thus it can’t be ignored that TGF-β also plays an important role in bone destruction in MM [73]. Overall, MM cells increase OC formation through activation of the RANKL-NF-κB signaling pathway and suppress bone formation through factors in the TME, such as DKK1, sclerostin, MMPs, activin A, integrins and TGF-β, etc. MMP-13 is the main OCs inducer while activin A is increased in MM patients with bone lesions and the inhibition of OBs differentiation and mineralization [76]. Also, VCAM-1 and α4β1 integrin not only enhance adherence of MM to BMSCs but also induce IL-6 and RANKL expression by BMSCs, stimulating OCs formation [4, 23]. Then, serum-increased levels of GDF-15 are associated with more advanced MM stages [77]. The interaction of OBs and myeloma cells can produce TGF-β to suppress the self-differentiation of OBs [53]. During osteoclastic bone resorption, TGF-β can be released from the bone matrix, inhibiting OB differentiation [58]. Also, the TGF-β-IL-11 axis can induce bone destruction [33]. Ashraf Z. Badros et al. found that in the serum, MM patients with bisphosphate (BP) related to osteonecrosis of the jaw (ONJ) (BRONJ) have significantly lower levels of TGF-β and VEGF over the study period [37]. Myeloma cells can upregulate the expression of TGF-β-associated kinase 1 (TAK1) [19]. Also, Jumpei Teramachi et al. reported that TAK1 combined with PIM2, the promotor of MM cell growth and survival, not only can be used as anti-apoptotic mediators in MM, but also be upregulated by inhibitors such as TGF-β and activin A for osteoblastogenesis [4, 13]. Additionally, intracellular adaptor proteins promote signal-induced activation of TAK1 to phosphorylate and activate IKK2, which is involved in bone destruction in the canonical NF-κB pathway [78]. However, various studies have reported that the binding of RANK to RANKL activates different signaling transduction pathways mainly involved in the dissolution and absorption of OCs [13]. In summary, it can be seen that the TGF-β signaling pathway, or combined with other pathways mainly inhibits the differentiation of OBs while the formation and increase of OCs mainly depend on other signaling pathways. Targeting TGF-β may alleviate bone destruction in MM patients, thereby improving quality of life and OS.

## Drugs targeting TGF-β and future perspectives

This review examines TGF-β effects on immunity and metabolism in MM, highlighting its role in suppressing immune responses. It explores how TGF-β interacts with different immune cell types in the TME and its influence on common TME features like hypoxia and acidification. Specifically, it discusses TGF-β’s impact on bone metabolism due to its relevance to bone pain in MM. However, the coordination between TGF-β and other components in the microenvironment remains unclear. Addressing this requires realistic MM models. Understanding these dynamics can inform the development of TGF-β-based immunotherapies for tumors thriving in TGF-β-rich environments.

TGF-β shapes the immune-suppressive TME in MM, fostering myeloma cell survival, invasion, and metastasis. It induces EMT in myeloma cells, enhancing stem-like properties, drug resistance, immunosuppressive ligand expression, and genomic instability. Given TGF-β’s pivotal role in MM and treatment challenges, therapies targeting TGF-β and its signaling pathways are actively researched and clinically assessed. According to the mechanism, these drugs mainly include (1) direct inhibition of synthesis of TGF-β by antisense oligonucleotide (ASOs); (2) using mAbs or soluble TGF-β Trap (TRAP) to block the interactions between TGF-β and its receptors; (3) using kinase inhibitors or interference with downstream of TGF-β adaptor for signal protein function to block TGF-β signaling pathway. YL-13,027, a novel inhibitor of TGF-βR1 that falls into the second category and developed by Shanghai Maya Li Pharmaceutical Co., LTD., is undergoing Phase I clinical trials. It shows potential for treating relapsed/refractory MM (R/RMM), particularly in cases with high TGF-βR1 expression on myeloma cells. However, the study of TGF-β’s dual role in MM is incomplete. TGF-β is present in cells throughout the body, regulating key physiological processes. Thus, its use may lead to adverse reactions, such as neurological disorders, reversible keratoacanthoma, excessive keratinization, and cytokine release syndrome. Additionally, diverse TGF-β downstream pathways and the lack of biomarkers for patient categorization and dosing optimization contribute to drug failures. Further research integrating bioinformatics, single-cell sequencing, and targeting agents is needed to develop effective TGF-β-related drugs. Finally, a series of scientific findings have shown that inhibiting the TGF-β signaling pathway may improve the therapeutic efficiency of PD-1/PD-L1 targeted drugs. Thus, they have chosen to combine the two therapies to improve clinical outcomes, which is likely to become the main direction to treat R/RMM in the future.

## Data Availability

No datasets were generated or analysed during the current study.
